# Early Intubation vs. Supportive Care in Management of Severe Blunt Chest Trauma; a Randomized Trial Study

**Published:** 2019-07-09

**Authors:** Mohammad Nasr-Esfahani, Amir Bahador Boroumand, Mohsen Kolahdouzan

**Affiliations:** 1 Department of Emergency Medicine, School of Medicine, Isfahan University of Medical Sciences, Isfahan, Iran. M_nasr@med.mui.ac.ir, ORCID: 0000-0002-5496-9170; 2 Department of Emergency Medicine, School of Medicine, Isfahan University of Medical Sciences, Isfahan, Iran. Abbg68@yahoo.com, ORCID: 0000-0001-5055-6881; 3 Department of surgery, Faculty of medicine, Isfahan university of medical sciences, Isfahan, Iran. kolahdouzan@med.mui.ac.ir, ORCID: 0000-0002-3095-6471

**Keywords:** Intubation, wounds and injuries, thorax, hospitalization, multiple trauma

## Abstract

**Introduction:**

Early intubation is one of the critical issues in patients with chest trauma. This study aimed to examine the effect of early intubation on outcomes of patients with severe blunt chest trauma.

**Methods:**

This *clinical trial* was performed on patients with blunt chest trauma referring to emergency department. Patients were randomly divided to intervention (early intubation) and control (supportive care) groups and the duration of hospitalization, complete recovery rate, laboratory changes, and in hospital mortality were compared between the two groups.

**Results:**

64 cases were divided into two equal groups of early intubation and control. There were no significant differences between two groups regarding age (p=0.36), sex (p=0.26), type of trauma (p>0.05), and comorbid diseases (p>0.05). The duration of hospitalization in the early intubation group was significantly lower than that of the control group (p = 0.01). 90.6% of those in early intubation group and 68.8% of those in the control group showed complete recovery (p = 0.03). There was no case of mortality in either group. There was a significant difference in venous blood pH between the groups at 6, 12, 18 and 24 hours after intubation (p < 0.05). Also, there was a significant difference in the HCO3 level at 6 and 12 hours after intubation (p <0.05).

**Conclusion:**

Early intubation is better than supportive treatment in patients with severe chest trauma because of a better complete recovery rate, lower duration of hospitalization, and better acid/base situation.

## Introduction

 *Severe blunt chest trauma* may causes rupture of the lung tissue, pulmonary contusion, intra-parenchymal hemorrhage, and alveolar collapse (1). Examination of chest injuries at the capillary level has shown that pulmonary contusion can lead to lung edema, alveolar edema, and even severe perivascular edema, resulting in the activation of inflammatory mediators, each of which causes lung tissue edema and inappropriate function of vascular permeability and changes in surfactant, even in areas that have not been traumatized (1-3).

Airway management is one of the first and most important principles of saving the lives of traumatic individuals, which should be done properly in people who are expected to have a respiratory problem (4). Failure to protect the airway is the most important cause of preventable mortality after a traumatic event (5).

The most important ways of protecting the respiratory tract are laryngoscopy and endotracheal intubation, which are the most reliable, safe and most commonly used methods for facilitating ventilation (6). Depending on the severity of chest trauma, 50-70% of people develop respiratory failure (7), and they need to be intubated. Given the importance of intubation in trauma patients, in 2002 the Eastern Association for the Surgery of Trauma (EAST) has defined indications for immediate intubation in traumatic individuals. These indications include: airway obstruction, reduced respiratory rate, severe hypoxemia, decreased consciousness and severe brain injury (Glasgow Coma Scale (GCS) ≤ 8), cardiac arrest, and severe hemorrhagic shock (8, 9).

Such indications are known as early and late indications of intubation. But, some of the intubations that can be performed shortly after the trauma based on the decision of the physician, or for other reasons indicative of the patient's need for intubation. Such indications, usually include damage to the face, changes in the level of consciousness, *difficulty breathing*, respiratory distress, intoxication, and preoperative control (8).

 A study by Trupka et al. showed that early intubation of trauma patients within 2 hours after injury is a useful and safe method that is capable of reducing post-traumatic organ failure and improving outcome (10). But, Sise et al. found no significant difference between the patients who underwent early intubation and the control group regarding the rate of hypotension, bradycardia, and aspiration (8). 

Considering the contradictory results presented in the studies and the importance of preserving airways in patients with chest trauma and pulmonary damage, this study aimed to compare the outcomes of early intubation versus supportive care in management of patients with severe blunt chest trauma. 

## Methods

***Study design and setting***

This *randomized clinical trial* was performed on patients with multiple traumatic injuries referring to emergency department (Level I) of Al-Zahra and Kashani Hospitals, Isfahan. Iran, from Jan 2016 to Dec 2018. The methodology of this research was approved by Ethics committee of Isfahan University of Medical Sciences (code=IR.MUI.REC.1397.074). This study is registered in Iranian registry of clinical trials database with IRCT20130311012782N31 code.

***Participants***

All adult (age > 18 years) multiple trauma patients with a trauma severity score of ≥ 5 based on Thoracic Trauma Severity (TTS) scoring system, with informed consent and agreement for participating were included. Patients who needed rapid-sequence intubation (less than 6 minutes after entering the emergency room), including severe head trauma patients with GCS ≤8, patients with respiratory distress with respiratory rate less than 9 and/or more than 30, airway obstruction, severe hypoxemia, cardiac arrhythmias, and severe hemorrhagic shock, as well as burns of more than 40%, severe burns of the face, *oropharynx* and *trachea*, and airway obstruction were not enrolled in the study.

***Intervention***

After the initial evaluation, clinical examination, and critical consideration by an emergency phycision, eligible patients undewent portable chest radiology, and the patients’ trauma severity scores were calculated using TTS system (11). Then patients were randomly divided into two parallel groups of early intubation (intervention) or supportive care (control) using random allocation software (block randomization method). 

After full respiratory and cardiac monitoring, early intubation cases underwent endotracheal intubation using rapid sequence intubation method. All intervention subjects were ventilated using synchronized intermittent mechanical ventilation (SIMV) mode, 8cc per kilogram tidal volume, 100% Fio2, and 12 times respiration per minute. Patients were extubated after one day (24 hours) of intubation, if they did not have the necessary contraindications.

Control group underwent routine supportive care such as, oxygen therapy with oxygen mask (100% oxygen with 8 to 10 liters per minute flow), head and neck positioning, pulse oximetry, and full respiratory and cardiac monitoring. 

Both groups received midazolam and fentanyl for relaxation and pain relief. All patients were managed by a senior emergency medicine resident under direct supervision of an emergency medicine specialist.

***Data gathering***

Demographic data, type of trauma, need for blood transfusion, and trauma severity based on TTS were recorded for all patients. Venous blood gas analysis (pH, Hco3, Pco2, PaO2), vital signs (systolic blood pressure, diastolic blood pressure, pulse rate, temperature), hemoglobin (Hb), and blood sugar (BS) were measured and recorded every three hours until six hours after entering the emergency room and then every 6 hours (for 24 hours in total) for both group. 

***Outcomes***

The duration of hospitalization (from admission to discharge from surgery department), complete recovery rate, laboratory and hemodynamic changes, and in hospital mortality were considered as measured outcomes. 

***Statistical analysis***

Considering 80% power and 95% confidence interval (CI), and the precision of estimation of 7% for standard deviation, minimum sample size was calculated to be 32 cases per group. Data were analyzed using SPSS version 20 software. Quantitative data were presented as mean and standard deviation, and qualitative data were indicated as percentage and frequency. Chi-square test was used to compare quantitative data between the groups. Independent t-test was used to compare the qualitative data between the groups. Also, repeated measures ANOVA was used to compare the changes in quantitative data at different times. A p-value less than 0.05 was considered as significant.

## Results

 ***Baseline characteristics of studied patients***

**Figure 1:**
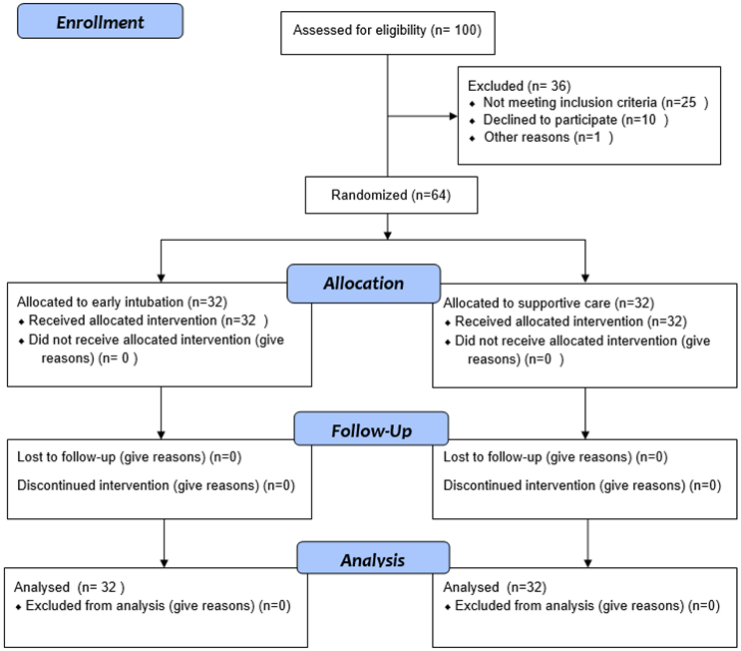
Flow diagram of patients enrollment

64 cases were divided into two equal groups of early intubation and control (figure 1). Table 1 compares the baseline characteristics of the studied patients. There were no significant differences between the two groups regarding age (p=0.36), sex (p=0.26), type of trauma (p>0.05), and comorbid diseases (p>0.05).

***Outcomes***

Tables 2 and 3 compare the studied outcomes between groups. The duration of hospitalization in the early intubation group was significantly lower than that of the control group (p = 0.01). 90.6% of those in the early intubation group and 68.8% of those in control group showed complete recovery (p = 0.03). There was no case of mortality in either group.

There was a significant difference in venous blood pH between the groups at 6, 12, 18 and 24 hours after intubation. Also, there was a significant difference in the HCO3 level at 6 and 12 hours after intubation (p <0.05).

**Table 1:** Baseline characteristics of patients in early intubation and supportive care groups

**Variable**	**Early intubation **	**Control **	**P**
**Age**			
Mean ± SD	42.03±16.76	45.28±19.36	0.36
**Sex**			
Male	21 (65.6)	25 (78.1)	0.26
Female	11 (34.4)	7 (21.9)	
**Comorbid disease**			
Diabetes	7 (21.9)	6 (18.8)	0.75
Hypertension	12 (37.5)	7 (21.9)	0.17
**Type of trauma**			
Bone fracture	25 (75.1)	22 (68.8)	0.57
Pneumothorax	23 (74.2)	21 (65.6)	0.45
*Hemothorax*	14 (43.8)	12 (37.5)	0.61
Free intra-*abdominal* fluid	5 (15.6)	4 (12.5)	0.71
**Trauma severity**			
TTS score	7.53±1.66	7.18±1.53	0.56

Data are presented as mean ± standard deviation (SD) or frequency (%). TTS: Thoracic Trauma Severity.

**Table 2:** Outcomes of patients in early intubation and supportive care groups

**Outcome**	**Early intubation **	**Control **	**P**
Duration of hospitalization	5.18±1.33	9.43±2.25	0.01
Complete recovery	29 (90.6)	22 (68.8)	0.03
Recovery with complications	3 (9.4)	10 (31.3)	0.01

Data are presented as frequency (%).

**Table 3:** Venous blood gas analysis, vital signs, glucose and hemoglobin changes among patients in early intubation and supportive care groups

**Variable**	**Early intubation**	**Control **	**P**
**pH**			
On arrival	7.30±0.09	7.18±0.13	0.21
3h	7.25±0.06	7.17±0.10	0.05
6h	7.38±0.09	7.23±0.17	0.006
12h	7.39±0.10	7.29±0.16	0.05
18h	7.40±0.07	7.36±0.15	0.009
24h	7.38±0.06	7.34±0.18	>0.001
**HCO3**			
On arrival	23.63±5.54	18.42±8.86	0.18
3	22.41±7.53	18.23±7.03	0.68
6h	23.80±3.46	21.34±7.91	>0.001
12h	24.76±3.18	26.21±8.19	>0.001
18h	25.32±5.76	26.89±7.51	0.17
24h	24.70±6.18	26.02±7.27	0.34
**PCO2**			
On arrival	50.05±16.73	47.41±19.17	0.76
3h	51.16±20.02	49.21±19.87	0.95
6h	49.18±13.52	40.49±9.14	0.02
12h	52.60±13.10	42.04±12.71	0.46
18h	45.90±9.78	42.69±10.25	0.57
24h	44.34±8.87	41.01±11.59	0.27
**Systolic blood pressure (mmHg)**			
On arrival	131.51±24.94	115.25±31.56	0.16
6h	120.96±18.89	116.25±24.66	0.58
12h	123.84±18.98	117.35±18.37	0.62
24h	130.43±18.36	122.01±21.84	0.62
**Diastolic blood pressure (mmHg)**			
On arrival	79.16±15.61	68.18±20.15	0.14
6h	77.40±13.53	70.21±14.08	0.84
12h	77.56±11.80	71.48±15.28	0.32
24h	81.20±11.24	75.76±14.32	0.35
**Pulse rate (/minutes)**			
On arrival	89.22±19.58	95.78±18.60	0.79
6h	88.06±15.16	93.37±22.03	0.14
12h	92.75±15.71	93.03±22.14	0.09
24h	89.06±13.79	91.61±19.02	0.10
**Temperature (c)**			
On arrival	37.12±0.48	37.09±0.57	0.74
6h	37.27±0.50	37.05±0.33	0.18
12h	37.32±0.47	37.20±0.47	0.42
24h	37.21±0.34	37.27±0.53	0.25
**Blood sugar (mg/dl)**			
On arrival	147.32±77.98	159.43±61.49	0.71
24h	163.38±112.76	167.31±115.29	0.70
**Hemoglobin (mg/dl)**			
On arrival	12.87±2.24	15.70±7.31	0.30
24h	14.20±5.81	13.59±2.18	0.28

## Discussion

 Based on the findings of this study, use of early intubation for patients with severe blunt trauma results in lower duration of hospitalization and better acid/base balances without any hemodynamic and laboratory impairment.

Recent studies have focused more on protecting the patient and using less risky methods in patients who do not have a clear indication for early intubation, while few studies have been done to discuss early intubation.

A study done by Sise and colleagues examined the indications for early intubation to evaluate the incidence and outcomes in 1,000 consecutive patients. They indicated that early intubation indications might change depending on the surgeon's view, and also no significant difference was found between the patients who underwent intubation and the control group for hypotension, bradycardia, trauma during intubation, and aspiration (8). The results of this study, which had a large sample size, are similar to our study as they considered early intubation to be practical and useful. 

Another study by Trupka et al showed that early intubation of injured patients within 2 h after trauma is a useful and safe method that is capable of reducing post-traumatic organ failure and improving outcome (10). 

As indicated by studies, one of the indications that affect early intubation, which is mostly dependent on the physician's opinion, is the patient's severe pain. It has been shown that early intubation of the patient due to severe pain can improve hospitalization outcomes and reduce postoperative complications (12). This factor can be controlled by administering appropriate analgesics to patients with trauma. In another study, among 120 intubated cases, half of them received strong painkillers, resulting in 3% of patients being subjected to intubation due to severe pain (13). 

The probability of developing respiratory infections after intubation or ventilator-associated pneumonia (VAP) is another issue that is considered about early intubations. However, our study did not indicate the incidence of this problem. Evans et al. (14) also examined prehospital intubation of patients with trauma and showed that early intubation was not associated with an increased risk of VAP. Also in a review article, rapid intubation was recommed for traumatic patients (15). 

The rate of on arrival PCO2 is critical in traumatic patients and explicitly affects the outcome of patients (16). Another study assessed 890 intubated patients and found that on arrival hypercapnia and hypocapnia worsen the results of hospitalization in those who are intubated (17). Of course, this study examined patients with head trauma, but, we can conclude that early intubation can be beneficial and lead to better regulation of blood gases and blood Pco2 in comparison with control group. 

Early intubation of trauma patients, based on the physician's opinion, is preferable to supportive care as it led to shorter hospitalization time, better recovery, and better VBG results.

## Limitations

The study limitations were small sample size and short duration of subsequent follow-up. It could nonetheless be used as a guide for other studies with larger sample sizes and human-financial resources. 

## Conclusion:

Based on the findings of this study, the use of early intubation for patients with severe blunt trauma results in lower duration of hospitalization and better acid/base balances without any hemodynamic and laboratory impairment.
